# Chalcone Derivatives: Promising Starting Points for Drug Design

**DOI:** 10.3390/molecules22081210

**Published:** 2017-07-25

**Authors:** Marcelo N. Gomes, Eugene N. Muratov, Maristela Pereira, Josana C. Peixoto, Lucimar P. Rosseto, Pedro V. L. Cravo, Carolina H. Andrade, Bruno J. Neves

**Affiliations:** 1 Laboratory for Molecular Modeling and Drug Design, Faculty of Pharmacy, Universidade Federal de Goiás, Setor Leste Universitário, Goiânia 74605-510, Brazil; marcelo13farma@yahoo.com.br (M.N.G.); andradech@yahoo.com (C.H.A.); 2 Laboratory for Molecular Modeling, Eshelman School of Pharmacy, University of North Carolina, Chapel Hill, NC 27955-7568, USA; murik@email.unc.edu; 3 Laboratório de Biologia Molecular, Instituto de Ciências Biológicas, Universidade Federal de Goiás, Goiânia 74001-970, Brazil; maristelaufg@gmail.com; 4 Programa de Pós-Graduação em Sociedade, Tecnologia e Meio Ambiente, Centro Universitário de Anápolis—UniEVANGÉLICA, Anápolis 75083-515, Brazil; josana.peixoto@gmail.com (J.C.P.); lucimar.pinheiro@yahoo.com.br (L.P.R.); pedrovcravo@gmail.com (P.V.L.C.); 5 GHTM/Instituto de Higiene e Medicina Tropical, Universidade Nova de Lisboa, 1349-008 Lisboa, Portugal

**Keywords:** natural products, chalcone derivatives, chalcone synthesis, molecular modification strategies, computer-assisted drug design

## Abstract

Medicinal chemists continue to be fascinated by chalcone derivatives because of their simple chemistry, ease of hydrogen atom manipulation, straightforward synthesis, and a variety of promising biological activities. However, chalcones have still not garnered deserved attention, especially considering their high potential as chemical sources for designing and developing new effective drugs. In this review, we summarize current methodological developments towards the design and synthesis of new chalcone derivatives and state-of-the-art medicinal chemistry strategies (bioisosterism, molecular hybridization, and pro-drug design). We also highlight the applicability of computer-assisted drug design approaches to chalcones and address how this may contribute to optimizing research outputs and lead to more successful and cost-effective drug discovery endeavors. Lastly, we present successful examples of the use of chalcones and suggest possible solutions to existing limitations.

## 1. Introduction

**Figure 1:**
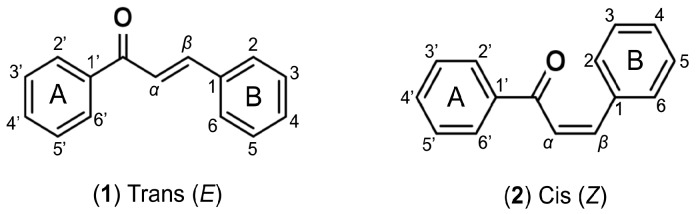
Structural and numerical representations of chalcone scaffold.

Chalcones, or 1,3-diphenyl-2-propen-1-ones, are one of the most important classes of flavonoids across the whole plant kingdom [1,2]. Chalcones are open-chain precursors for biosynthesis of flavonoids and isoflavonoids and occur mainly as polyphenolic compounds whose colour changes from yellow to orange [3]. They exist as either trans (*E*, **1**) or cis (*Z*, **2**) isomers having two aromatic rings that are joined by a three-carbon α,β-unsaturated carbonyl system (Figure 1). In most cases, the *E* isomer is more stable from the perspective of thermodynamics, which makes it the predominant configuration among the chalcones. The configuration of the *Z* isomer is unstable due to the strong steric effects between the carbonyl group and the A-ring [4].

**Figure 2:**
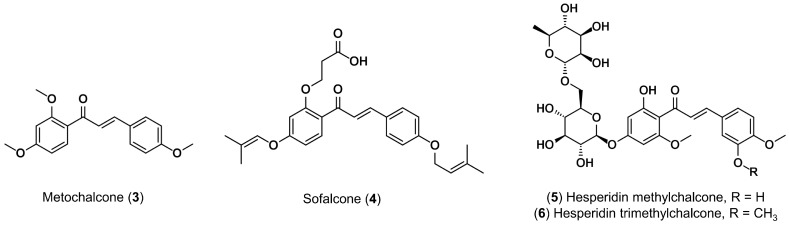
Chemical structures of approved and clinically tested chalcones.

The chemistry of chalcones remains a fascination among researchers in the 21st century due to the large number of replaceable hydrogens that allows a large number of derivatives and a variety of promising biological activities to be generated, e.g., anti-inflammatory [5], anti-gout [6], anti-histaminic [7], anti-oxidant [8], anti-obesity [9], anti-protozoal [10], hypnotic [11], anti-spasmodic [12], etc. Importantly, two chalcones have been used in clinical practice (Figure 2). Metochalcone (**3**) was approved as a choleretic drug [2], and sofalcone (**4**) as an anti-ulcer agent that increases the amount of mucosal prostaglandin, conferring a gastroprotective effect against *Helicobacter pylori* [13]. In addition, clinical trials have shown that hesperidin methylchalcone (**5**, tested for chronic venous lymphatic insufficiency) [14,15], and hesperidin trimethylchalcone (**6**, evaluated for trunk or branch varicosis) [16] led to the relieving of symptoms, reached reasonable plasma concentrations, and were well-tolerated.

Despite medicinal applications of chalcones, their wide bioactivity spectrum indicates a potentially promiscuous target profile, which presents a challenge for clinical development [17]. This is largely attributable to the electrophilic nature of the α,β-unsaturated carbonyl system. This moiety is capable of forming irreversible bonds with biological macromolecules, resulting in a number of toxic effects, such as allergenic reactions, carcinogenicity, and mutagenicity [18]. On the other hand, this reactivity may be affected both by the decoration of the aromatic rings, and also, even more effectively, by α-X-substitution of the double bond of the enone system [19]. Therefore, the design and synthesis of new analogs are particularly important for the future development of clinically useful chalcone derivatives.

Here, we aimed to review recent data on computer-assisted design and synthesis of new chalcone derivatives with improved pharmacodynamics, pharmacokinetics, and toxicological profiles; major challenges in the field; and possible solutions to existing pitfalls.

## 2. Synthesis of Chalcone Scaffolds

Chalcones have a simple chemistry which enables a multiplicity of substitutions with easy synthesis. Currently, a variety of methods and schemes are available for the synthesis of chalcone derivatives. In each of these methods, the most important part is condensation of two aromatic systems (with nucleophilic and electrophilic groups) to yield the chalcone scaffold. Despite the multiplicity of substitutions allowed, we describe below the reaction scheme using the standard scaffold of chalcones (1,3-diphenyl-2-propen-1-one).

### 2.1. Claisen-Schmidt Condensation

**Scheme 1:**
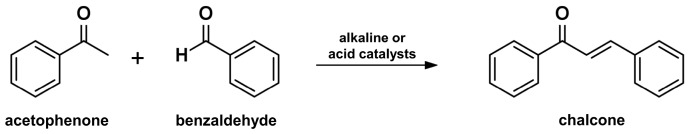
The Claisen-Schmidt condensation.

Amongst all methods, the Claisen-Schmidt condensation (Scheme 1) is one of the most common. In this reaction, chalcones are formed by condensation of benzaldehyde and acetophenone derivatives in the presence of alkaline or acid catalysts in liquid solvent at 50–100 °C for several hours [20,21,22]. The conventional Claisen-Schmidt reaction is typically carried out in the liquid phase, but certain reactions can take place in the solid phase (e.g., acetophenone derivatives are primarily bound to the resin and then treated with benzaldehyde derivatives) [22] or solvent-free phase (e.g., condensation in the presence of catalyst triazabicyclodecene) [23]. In addition, the use of microwaves in liquid and solvent-free Claisen-Schmidt reactions reduces synthesis time and yields good amounts of chalcones [24,25].

### 2.2. Carbonylative Heck Coupling Reaction

**Scheme 2:**
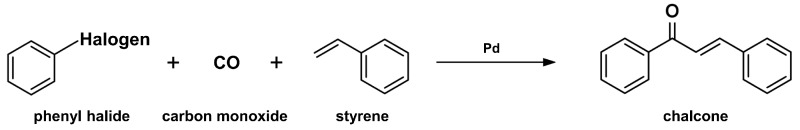
Carbonylative Heck coupling reaction.

In the carbonylative Heck coupling reaction (Scheme 2), chalcones are synthesized by carbonylative vinylation of phenyl halide with styrene in the presence of carbon monoxide and using palladium (Pd) as catalyst [26].

### 2.3. Coupling Reaction

**Scheme 3:**
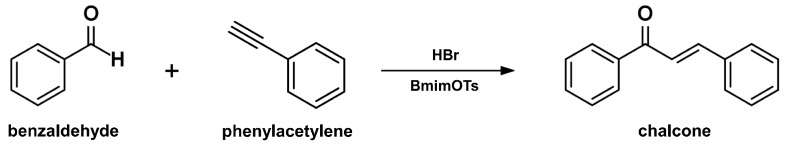
Coupling reaction.

Chalcones also are synthesized by a coupling reaction (Scheme 3) between benzaldehyde and phenylacetylene in the presence of HBr and ionic liquids, such as 1-butyl-3-methyl-1*H*-imidazolium 4-methylbenzenesulfonate (BmimOTs) for 12 h at 100 °C [27].

### 2.4. Sonogashira Isomerization Coupling

**Scheme 4:**
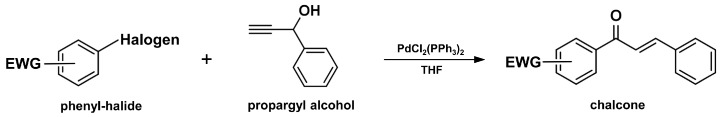
Sonogashira isomerization coupling. EWG: electron withdrawing group.

In the Sonogashira isomerization coupling reaction (Scheme 4), chalcones are synthesized through a reaction between the equimolar concentration of electron-deficient phenyl-halide and propargyl alcohol employing microwave irradiation and using PdCl_2_(PPh_3_)_2_ as catalyst and THF as solvent [28,29].

### 2.5. Continuous-Flow Deuteraction Reaction

**Scheme 5:**
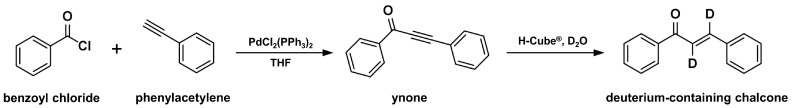
Continuous-flow deuteraction reaction.

In the continuous-flow reaction (Scheme 5), ynones are initially prepared by literature procedures through the coupling of benzoyl chlorides with phenylacetylenes under Sonogashira conditions (see above). Then, deuterations are carried out in an H-Cube^®^ system with replacement of the H_2_O hydrogen source to D_2_O deuterium source [30,31].

### 2.6. Suzuki–Miyaura Coupling Reaction

**Scheme 6:**
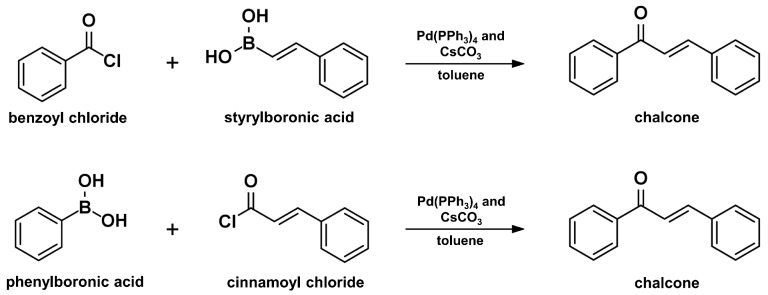
Suzuki–Miyaura coupling reaction.

In the Suzuki–Miyaura coupling reaction (Scheme 6), chalcone synthesis involves the coupling of benzoyl chloride with styrylboronic acid employing Pd(PPh_3_)_4_, CsCO_3_ and anhydrous toluene or coupling of phenylboronic acid with cinnamoyl chloride employing Pd(PPh_3_)_4_, CsCO_3_, and anhydrous toluene [32].

### 2.7. One-Pot Synthesis

**Scheme 7:**
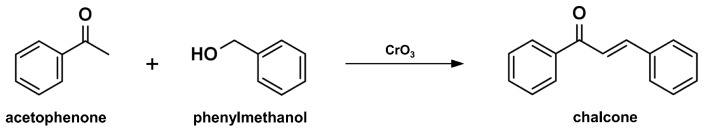
One-pot synthesis of chalcones.

The one-pot synthesis (Scheme 7) is a simple, yet efficient green method which allows synthesis of chalcones in just one reactor. This method provides several advantages, such as increased reaction efficiency, and avoidance of the lengthy purification process of the intermediate chemical compounds, thereby saving resources and time [22]. The reaction consists of a mixture of phenylmethanol and acetophenone in the presence of the oxidizing agent CrO_3_. In this reaction, CrO_3_ plays the vital role of generating the benzaldehyde from phenylmethanol, which further reacts with the acetophenone to produce the desired chalcone [22].

### 2.8. Solid Acid Catalyst Mediated Reaction

**Scheme 8:**
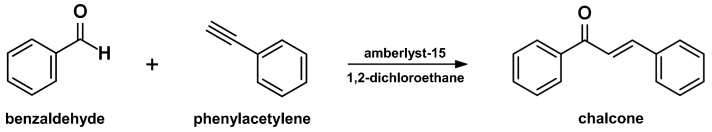
Solid acid catalyst mediated synthesis.

Chalcones have also been synthesized by employing a heterogeneous solid acid catalyst (Scheme 8). The reaction consists of the addition of an equimolar quantity of benzaldehyde and phenylacetylene in 1,2-dichloroethane solvent irradiated in a microwave and employing ion-exchange resin amberlyst-15 as heterogeneous solid acid catalyst [33].

## 3. Design of New Chalcone Derivatives

During the early steps of drug discovery projects, an intensive search ensues to find pharmacologically active small molecules with acceptable pharmacokinetic and toxicological properties, which are candidates for preclinical and clinical development [34]. The activity of a molecule can be correlated to its structure in terms of the lipophilic, electronic, and steric features of its functional groups. Hence by investigating the proper functional group, one can govern the biological pattern avoiding the occurrence of complications in pre-clinical and clinical assays. The schematic representation of the nature of such bioactive functional groups along with their interatomic distances is known as pharmacophore. Once such a pharmacophore is identified, molecular modification strategies can be explored to promote chemical alterations in the chalcone structure which lead to improvements in its physicochemical properties and biological profile. Among molecular modification strategies used, bioisosterism, molecular hybridization, and drug latentiation are the most widely used examples. We address each of these strategies below, highlighting their application(s) in the design of new chalcone derivatives. Lastly, we want to emphasize that chalcones, as perspective agents against cancer, bacteria, etc., were strongly underestimated because of the PAINS filtering [35]. Recently, several studies have demonstrated the inability of PAINS alerts to filter-out promiscuous compounds and their oversensitivity, i.e., rejection of many of non-promiscuous compounds [36,37]. Removal of PAINS filters from the pipeline and their substitution by orthogonal assays [37,38] will greatly increase the attractiveness of chalcones as potential starting points in drug discovery.

### 3.1. Bioisosterism

The term bioisostere was introduced by Harris Friedman in 1950. Recognizing the usefulness of isosterism to design bioactive molecules, Friedman defined bioisosteres as compounds which fit the broadest definition for isosteres and have a similar type of biological activity, either through agonist or antagonist actions [39,40]. This definition has been further expended by Alfred Burger as compounds or groups that possess near-equal molecular shapes and volumes, similar physicochemical properties, and which exhibit approximately the same distribution of electrons [40,41]. Today, the design of bioisosteres frequently introduces structural changes that, depending on the context, can be beneficial or deleterious. Electronic density, steric effects, polarizability, dipole, Log*P* and Log*D*, maintenance of intermolecular interactions, molecular conformation, Log*S*, and p*K*_a_ potentially play key contributing roles in molecular recognition and mimicry [42,43].

**Figure 3:**
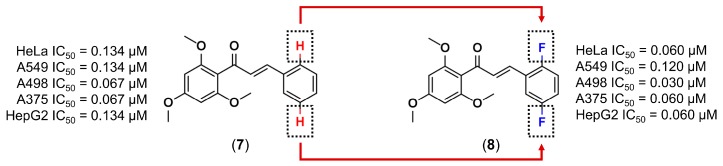
Bioisosterism represented by replacing the hydrogen (red) with a fluorine (blue) [45].

There may be innumerous reasons for the use of bioisosterism to design new chalcone derivatives, including the necessity to improve pharmacological activity, gain selectivity for a particular receptor or enzyme with simultaneous reduction of certain adverse effects, or even for optimizing the pharmacokinetics of the lead candidate [39]. For example, substituting a hydrogen atom by fluorine represents the most conservative example of bioisosterism given the similarities in steric parameters (van der Waal’s radii being 1.2 and 1.35 Å, respectively), but there are circumstances in drug design where this change can offer a significant advantage [40]. The influence of the electron-withdrawing effect such as fluorine substitution (fluorine being the most electronegative element in the periodic table) is often the basis for the major differences in the biological properties of compounds. Fluorine affects the bonding interactions and metabolic stabilities of compounds and has an impact on their physical features and selective reactivity [44]. Burmaoglu and colleagues [45] synthesized a series of bioisosteric molecules with anticancer activity, starting from a 2′,4′,6′-trimethoxy-chalcone (**7**) structure. Potent anticancer effects were reported for some of these bioisosteres. Of particular interest was derivative **8** (Figure 3), displaying potencies (IC_50_) ranging from 0.030 to 0.120 μM on a panel of cervical cancer (HeLa), lung adenocarcinoma epithelial (A549), renal cancer (A498), skin malignant melanoma (A375), and hepatocellular carcinoma (HepG2) cell lineages [45].

**Figure 4:**
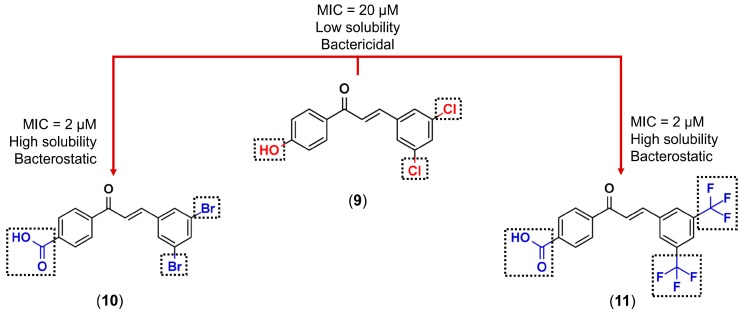
Bioisosterism represented by the replacing the hydroxyl and chloride groups (red) with a carboxylic acid and hydrophobic groups (blue) [46].

Another fine illustration of bioisosteric replacement has been documented for a series of antibacterial chalcones designed by Nielsen and colleagues [46]. Authors showed that the 4′-hydroxy group of the A-ring in combination with lipophilic substituents in the B-ring was essential for the activity of chalcones over *Staphylococcus aureus*. However, the modest antibacterial potency and the low water solubility have limited the usefulness of these compounds. Considering this, the hydroxyl group of the 4′-hydroxy-2,4-dichloro-chalcone (**9**) was substituted by a carboxylic acid group while halogens were substituted by another hydrophobic group (Figure 4). The hydroxyl does not resemble carboxylic acid in terms of size or as a strong electron-donating group, but total energy of H-bonding interactions and dramatic modification of the p*K*_a_ confers to this group greater solubility. The substituents in the B-ring were systematically changed to obtain the most diverse set of analogues. The substituents were selected based on their size, lipophilicity, and electronic properties. Following these conditions, two bioisostere chalcones (**10** and **11**) showed minimum inhibitory concentrations (MICs) of 2 μM, low cytotoxicity (>100 µM) against mammalian cells, and markedly improved solubility. Furthermore, the mechanism of action of **10** and **11** was different, as they were bacteriostatic, while the hydroxy chalcones were bactericidal. This might be an advantage, as it is believed that bactericidal compounds are far more toxic to mammalian cells than bacteriostatic compound [46].

**Figure 5:**
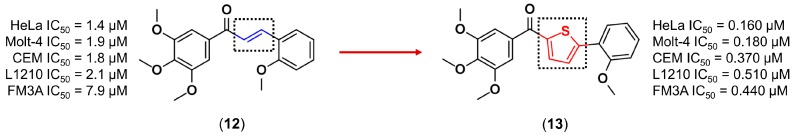
Bioisosterism represented by the replacing the double bond of the enone (blue) with a thiophene (red) [47].

The replacements where cyclic functional moiety mimics a noncyclic group sterically or electronically also result in the retention of biological activity. Here the ability of the structure to hold the critical functionality in a spatial configuration could be essential for activity or selectivity. For example, Romagnoli and colleagues [47] synthesized a series of thiophene diarylchalcones with anticancer activity, in which the double bond of the enone system was embedded within a thiophene ring (see Figure 5). The synthesized compounds were found to inhibit the growth of several cancer cell lines at nanomolar to low micromolar concentrations. The replacement of the double bond with a thiophene increases anticancer activity and must not significantly alter the relative conformation of the chalcone derivate **12**. Of particular interest was derivative **13**, showing IC_50_’s ranging from 0.160 to 0.510 μM on a panel of HeLa, human T-lymphocyte (Molt-4 and CEM), murine leukemia (L1210), and murine mammary carcinoma (FM3A) cell lineages [47].

### 3.2. Molecular Hybridization

The hybridization of biologically active molecules, based on the combination of complementary pharmacophoric features of two or more known bioactive compounds, which, through adequate fusion, lead to the design of new hybrid architectures that maintain pre-selected characteristics of the original templates, is a powerful strategy for drug design. There are two main ways through which hybrid molecules can be constructed. The first method combines two or more entire chemical structures together using a linker chain, whilst the second technique directly merges pharmacophoric moieties of different compounds [48,49]. In this context, to improve the efficacy of some chalcones in the treatment of diseases involving dual- or multi-target approaches, molecular hybridization has been investigated for the design of new ligands or prototypes.

**Figure 6:**
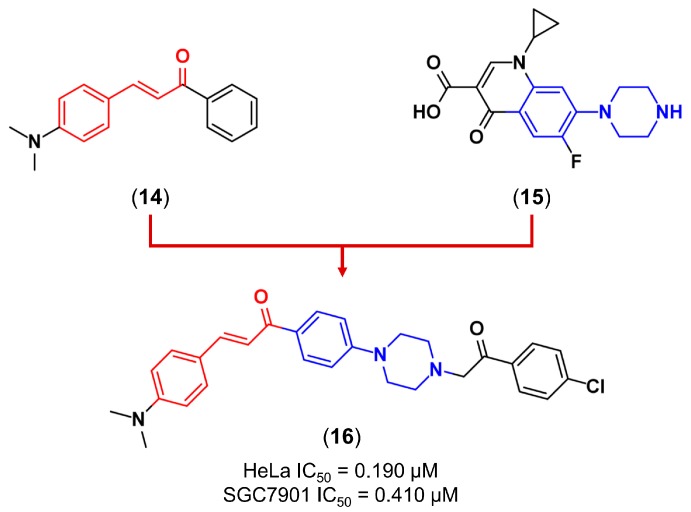
Molecular hybrid obtained from the combination of chalcones (red) and *N*-aryl piperazine moiety (blue) [50].

Mao and colleagues [50] designed and synthesized a series of compounds that have a chalcone derivate (**14**) and an *N*-aryl piperazine moiety (see ciprofloxacin structure (**15**)) in one frame and evaluated them for anticancer activity (Figure 6). Ciprofloxacin is a well-known inhibitor of human DNA topoisomerase II that exhibits potent in vitro anticancer activity against different cell lineages [51,52,53,54] while chalcones are known to inhibit several anticancer targets, including thioredoxin reductase [45], and tubulin polymerization [55,56]. Therefore, hybrid molecules that contain *N*-aryl piperazine and chalcone moieties are expected to hold improved activities. As a result, synergic anticancer effects were reported for some of these hybrids. Hybrid **16** displayed the highest activity against cervical cancer (Hela) and gastric cancer (SGC7901) cells with IC_50_ of 0.190 µM and 0.410 µM, respectively, in comparison to 20 µM and 12 µM for the positive control cisplatin [50].

**Figure 7:**
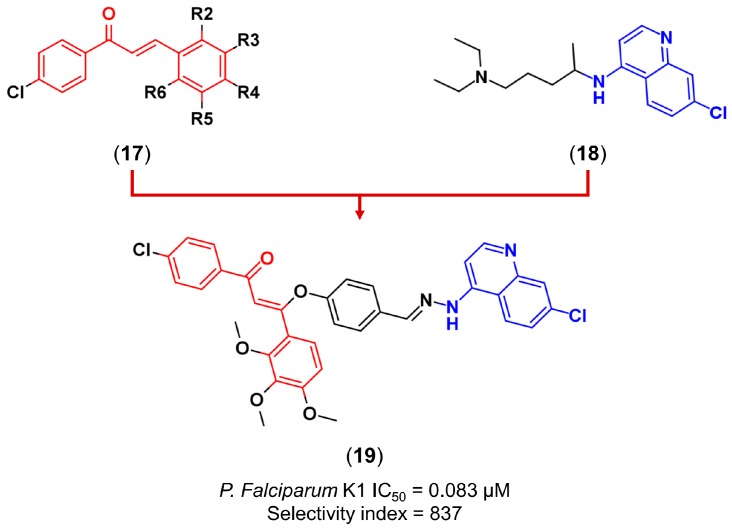
Molecular hybrid obtained from a combination of 4'-chlorochalcone (red) and chloroquine (blue) [57].

A series of hybrids made of 4′-chlorochalcone (**17**), and the antimalarial drug chloroquine (**18**) were designed and tested for their antimalarial efficacy against chloroquine-resistant strain (K1) of *Plasmodium falciparum* [57] (see Figure 7). Chloroquine interferes with the parasite detoxification process by inhibiting heme transformation into hemozoin. The accumulation of heme causes severe damage to the parasite, leading to its death [58,59]. The antimalarial property of chalcone derivatives is derived from their ability to inhibit the parasitic cysteine proteases, such as falcipain. These proteases catabolize globin into small peptides within the acidic food vacuole of the intra-erythrocytic malaria parasite [60]. In summary, a series of hybrids were synthesized with different substitution patterns on the chalcone phenyl ring B. The hybrids showed low cytotoxicity in epithelial kidney cells (Vero, selectivity index (SI) >559) and enhanced antimalarial activity as compared to chloroquine (IC_50_ > 0.463 µM) over the resistant strain of *P. falciparum* (IC_50_ ≤ 0.315 µM). The most effective hybrid **19** possessed IC_50_ of 0.083 µM and selectivity index of 837. The reference antimalarial drug chloroquine showed IC_50_ higher than 0.463 µM [57].

**Figure 8:**
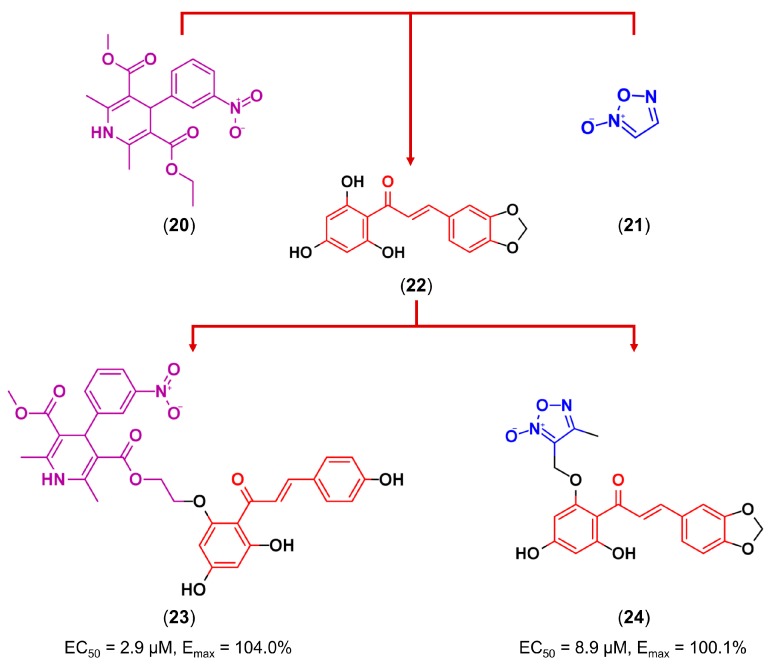
Molecular hybrids obtained from combination of well-known vasodilators (nitrendipine or furoxan, highlighted by purple and blue colors, respectively) and chalcones [61].

Dong and colleagues [61] combined nitrendipine (**20**) or furoxan (**21**) to the chalcone scaffold (**22**, see Figure 8). Nitrendipine is a well-known calcium channel blocker [62,63] while furoxan acts as a nitric oxide (NO) donor [64,65]. In addition to traditional vasodilators, the antioxidant properties of chalcones were also demonstrated to be beneficial in the treatment of cardiovascular diseases [66]. Two series of hybrids were made in which the 1,4-dihydropyridyl or furoxan moiety is located at the 2′-position of the chalcone scaffold. Many of the hybrids exhibited moderate to excellent vasorelaxant activities. Amongst the nine hybrids made, compounds **23** (EC_50_ = 2.9 μM, E_max_ = 104%) and **24** (EC_50_ = 8.9 μM, E_max_ = 100%) were the most active. The positive control quercetin showed EC_50_ of 244 µM [61].

**Figure 9:**
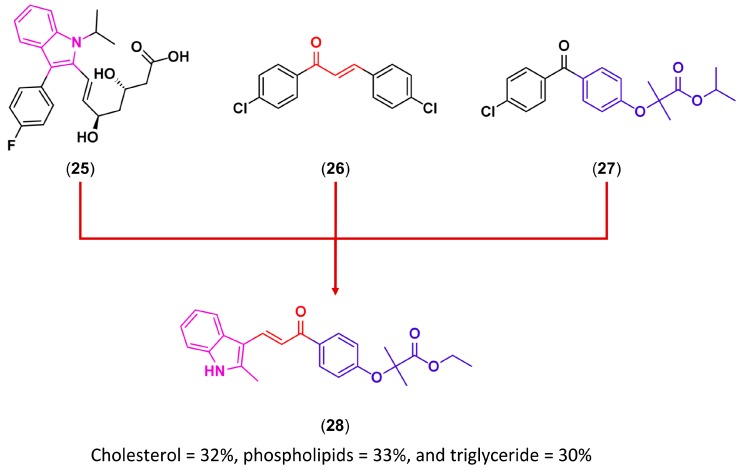
Molecular hybrids obtained from a combination of Fluvastatin (purple), fenofibrate (blue), and chalcones (red) [67].

A series of hybrids bearing an indole ring system associated to chalcone fibrate with variable substitution patterns were reported by Sashidhara and colleagues [67] as lipid-lowering agents (see Figure 9). Indole-based compounds have been a subject of intense investigations for their antihyperlipidemic activity [68]. Fluvastatin (**25**), compound with indole moiety, was the first synthetic 3-hydroxy-3-methylglutaryl coenzyme A reductase inhibitor (statin) and is used in the management of dyslipidemia in the primary and secondary prevention of cardiovascular disease [69,70]. In addition, a recent literature survey revealed that chalcone derivatives, such as 4′,4-dichlorochalcone (**26**), exhibited potential hypolipidemic activities [71]. Fibrates are selective agonists of the peroxisome proliferator-activated receptor gamma (PPARγ). Drugs such as fenofibrate (**27**) lower triglyceride levels and increase high-density lipoprotein (HDL) levels in hyperlipidemic patients and reduce the risk of coronary heart disease [72,73]. These interesting biological properties led authors to link indole, chalcone, and fibrate moieties with the aim of obtaining potent antidyslipidemic agents. One of the best hybrids, compound **28**, was shown to decrease the concentration of cholesterol, phospholipids, and triglyceride of hyperlipidemic rats by 32%, 33%, and 30%, respectively [67].

### 3.3. Drug Latentiation

Undesirable properties, including poor aqueous solubility, chemical instability, insufficient oral or local absorption, low half-life, fast pre-systemic metabolism, local irritation, and toxicity are typical problems involved in drug design. For these reasons, drug latentiation presents an effective strategy to improve these properties. The term latentiation may be defined as the chemical union between a compound and a transporter group (e.g., ester, amide, carbamate, carbonate, ether, imine, and phosphate), normally by means of a covalent labile bond, giving rise to the classical concept of a prodrug. It is a temporarily inactive compound that undergoes in vivo biotransformation through chemical or enzymatic cleavage, enabling the delivery of the active molecule at efficacious levels. Consequently, it can facilitate the accumulation of a drug at the site of action and improve safety [74,75].

**Figure 10:**
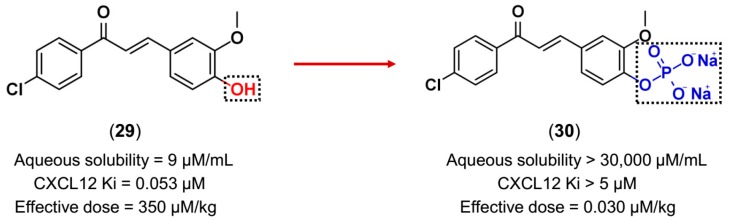
Latentiation represented by the replacing the hydroxyl (red) with a phosphate (blue) [77].

For example, some chalcone derivatives, such as compound **29**, prevents inflammatory reactions through neutralization of the CXC motif chemokine ligand 12 (CXCL12, inhibitory constant (Ki) of 0.053 µM) and prevents it from acting on the CXCR4 and CXCR7 receptors, although poor solubility in water limits their use [76]. To overcome this drawback, Gasparik and colleagues [77] synthesized a series of chalcone prodrugs with different functional groups, such as phosphate, l-seryl, and sulfate. The chalcone prodrug with a phosphate substituent (**30**, Figure 10) showed a greater increase in solubility, to at least 3000 times greater solubility than the parent chalcone. The compound behaves as a prodrug, remaining inactive in in vitro assays, but when cleaved in vivo into **29**, inhibits eosinophil recruitment in the airways by ≥50% at a dose as low as 0.030 µM/kg and without any signs of toxicity [77].

**Figure 11:**
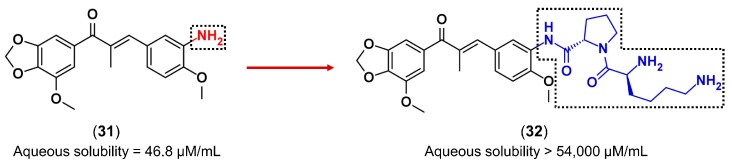
Latentiation represented by the replacing the amine (red) with an l-Lysine-l-Proline (blue) [78].

Amino acids were also used to improve the physicochemical properties of chalcone derivatives. For example, Canela and colleagues [78] investigated the tubulin-binding, vascular targeting, anti-tumor and anti-metastatic activities of a new series of chalcone derivatives. The best compound of this series (**31**) inhibited the proliferation of endothelial (HMEC-1, microvascular endothelial cell line-1; and BAEC, bovine aortic endothelial cells) and tumor (B16-F10.luc2, melanoma cells expressing firefly luciferase 2; Cem; and HeLa) cell lines with IC_50_ concentrations between 0.001 and 0.004 µM, which is slightly better than the reference compound colchicine. However, poor aqueous solubility (0.016 mg/mL) precluded in vivo evaluation. Therefore, the authors synthesized prodrugs of compound **31** by conjugation with amino acids (Figure 11). The l-Lysine-l-Proline derivative **32** was around 2000-fold more soluble than the parent compound and was effective in inhibiting tumor and endothelial cell proliferation. In addition, incubation of prodrug **32** in human serum or murine liver extract showed an efficient release of **31**. The prodrug candidate **32** also showed potent in vivo anticancer activity in melanoma (10 mg/kg) and breast cancer models (15 mg/kg) by causing rapid intratumoral vascular shutdown and massive tumor necrosis [78].

## 4. Computer-Assisted Drug Design (CADD)

The concept of design, synthesis, and biological evaluation forms a central pillar of the lead discovery process and provides the basis for our understanding of the underlying structure–activity relationships (SAR) [34]. However, synthesis and bioassays are laborious and the chance of getting false positives is high. Although expert biological knowledge is indisputably important for successfully guiding drug discovery projects, CADD approaches, as well as the availability of supercomputers, parallel processing, and the graphics processing unit (GPU), have been playing a pivotal role on prioritizing compounds for synthesis and/or biological evaluation. As no compounds need to be synthesized or tested before computational studies, CADD represents a time-, labor-, and cost-effective strategy to obtain lead compounds in the early stages of drug discovery [79,80,81]. In the subsequent sections, we review current developments in CADD, highlighting the main strategies and pitfalls that have solid implications in the medicinal chemistry of chalcone derivatives.

### 4.1. Structure-Based Drug Design (SBDD)

In SBDD, 3D structural information of a macromolecule is used to design or evaluate ligands based on their predicted interactions and affinity with protein binding sites. Thus, identification of a valid biochemical target and the acquisition of its structural information are the first vital steps in SBDD. Researchers from computational and structural biology aided in the generation of thousands of structures with the use of homology modeling, X-ray crystallography, cryo-electron microscopy (EM), and nuclear magnetic resonance (NMR) [81,82,83]. Currently, more than 13,000 3D structures have been experimentally solved and stored in the Protein Data Bank (PDB), leading to attractive opportunities for the application of SBDD strategies [84].

SBDD can be divided into virtual screening (VS) and de novo approaches. In VS large chemical libraries are processed to search for compounds that possess complementarities toward the targets. De novo design exploits information from the 3D biomacromolecule to find small fragments that match well with the binding site. These fragments should be linked, providing a structurally novel ligand that can be synthesized for further in vitro assays [85,86]. However, because of its knowledge-based characteristic, SBDD strongly depends on the amount and the quality of information available about the system under investigation. Regardless of what kind of protein will be employed as a molecular target, important issues, such as the suitable assignment of protonation states [87,88], solvation [89,90], and protein flexibility [91,92], must be considered. Protein-ligand docking, molecular dynamics simulations, and structure-based pharmacophores are the main SBDD tools. Basic principles, advantages, and disadvantages of these tools are discussed below.

#### 4.1.1. Protein-Ligand Docking

Protein-ligand docking is the most extensively used SBVS method. It predicts possible binding modes of a compound or fragment in a target binding site and estimates affinity based on its intermolecular interactions in the binding pocket. Docking is often carried out in two parts. In the first part, the ligand is placed inside the binding site in different orientations and conformations using a search algorithm to facilitate the identification of the binding mode. Then, a scoring function ranks the different poses of the ligand that are generated by the search algorithm and orders them by a score. These scores are features that aid in investigating the interactions between the small molecule and the biological target, thereby providing context about biological activity [82,93,94]. The discussion about search algorithms and scoring functions is beyond the scope of this review, but detailed information can be obtained elsewhere [95,96,97,98,99].

Although the concept of docking seems to be precise, it remains a major challenge of this approach. Usually, the scoring functions are found to have limited accuracy in the ranking of compounds and sometimes cannot distinguish between active and inactive molecules. In addition, the insertion of protein flexibility to some active residues can help in identifying new hits with better molecular complementarity, when compared with traditional rigid docking. However, it requires more complex calculations because the proteins of high conformational energy score would result in idealistic binding mode [85]. Consequently, some streamlined strategies have been developed to solve these methodological limitations. To incorporate protein flexibility, ensemble methods make use of multiple input conformations of a target using a set of different 3D structures experimentally determined (X-ray crystallography or NMR) or computationally produced using molecular dynamics simulations (see below). Instead of single rigid structure, carefully chosen 3D conformations of the target can be used to represent conformational changes in the protein backbone and/or side chain [100,101]. On the other hand, averaging the results from two or more scoring functions and forming a consensus has proved to be more beneficial in scoring and ranking compounds [102]. In addition to consensus scoring, post-processing techniques such as the molecular mechanics Poisson–Boltzmann surface area methods can be used to better estimate the free binding energies [103,104].

#### 4.1.2. Molecular Dynamics (MD) Simulations

MD allows the prediction of the time-dependent behavior of a molecular system. It has been particularly useful in the structural refinements of post-docking complexes, such that the complementarity between the ligand and the receptor is enhanced in the complex state, allowing better complementarities. The knowledge of MD functionalities of proteins is important in order to understand several key aspects of SBDD, such as ligand–protein interactions, as well as exploring the energy landscapes of proteins and identifying their physiological conformations, which are, in many cases, not even accessible through experimental techniques [105,106]. Methodologically, MD regards atoms as solid spheres and the bonds connecting them as springs. The trajectories of atoms and molecules are determined by numerically solving Newton’s equations of motion for a system of interacting particles, where forces between the particles and their potential energies are calculated using force fields with predefined parameters. A force field is a mathematical expression describing the dependence of the energy of a system on the coordinates of its atoms. This includes the possible nonbonded interactions (Coulomb potentials and van der Waals potentials) and bonded (bonds, angles, and dihedrals) terms between the different atoms in a 3D macromolecule [105,106]. Several force fields are available for MD simulations, such as GROMOS [107], CHARMM [108], and AMBER [109]. Most of these methods have different functional forms to treat MD simulations, which makes it difficult to apply parameters from one force field to another.

#### 4.1.3. Structure-Based Pharmacophores (SBPs)

In addition to docking, 3D structural information may be used to derive pharmacophore hypotheses. They are defined by the International Union of Pure and Applied Chemistry (IUPAC) as “an ensemble of steric and electronic features that is necessary to ensure the optimal supramolecular interactions with a specific biological target and to trigger (or block) its biological response” and can be used to screen a compound database [110]. Here, SBPs describe the spatial arrangement of essential ligand-macromolecule interactions directly from the 3D structural data. This approach determines chemical features based on complementarities between a ligand and its binding site. Nonetheless, the selection of features in SBPs is a more complex effort than in ligand-based, since there are more possibilities of conformational and spatial states [111,112].

Typically, SBPs can be created using ligand-macromolecule complexes (holo) or using ligand-free macromolecules (apo). The generation of models using holo structures allows complete exploration of the ligand interactions with the binding site, and the inclusion of shape and volume information derived directly from the structural data [111,113]. In the absence of a ligand in the binding site, pharmacophores can be obtained using functional groups or small fragments, also referred to as molecular probes, to map possible interaction sites or hot spots within a binding site. Selected hot spots can then be converted into pharmacophoric features to generate a structure-based pharmacophore model [113].

### 4.2. Ligand-Based Drug Design (LBDD)

In some cases, usually when data pertaining to the 3D structure of a target protein are not available, CADD can instead be based on processes using the known ligands of a target protein or known active compounds in phenotypic assays as starting points. This approach is known as LBDD [114,115]. Similarity search, ligand-based pharmacophores, and quantitative structure-activity relationship (QSAR) analysis are the most popular methods in the LBDD process (described below).

#### 4.2.1. Similarity Search

The major principle of medicinal chemistry is the assumption that structurally similar compounds exhibit similar biological activities. Based on this premise, similarity search techniques have been applied to represent chemical structures to allow rapid structural comparison in an effort to identify structurally similar compounds or to cluster collections based on structural similarity. Methodologically, both query ligand and the database of compounds could be represented and further compared using molecular fingerprints, a string made up of binary digits that account for the presence (1) or absence (0) of representative fragments or atoms in the chemical structure. These fingerprints vary greatly in length and complexity ranging from simple topological representations to complicated multi-point 3D pharmacophore arrangements [116,117]. Then, bit vector similarity must be expressed in a way that can be quantified. The most used metric to explore similarity is the Tanimoto coefficient, which is equal to the number of common bits set to 1 in both fingerprints divided by the total number of bits set to 1 between both fingerprints [118]. It assumes that two structures can be considered similar if this coefficient is higher than 0.85.

#### 4.2.2. Ligand-Based Pharmacophores (LBPs)

The pharmacophore approach aims to identify containing different scaffolds, but with a similar 3D arrangement of key interacting functional groups. LBPs identify key common features (e.g., hydrogen bond donors or acceptors, aromatic rings, partial charges, and hydrophobicity) and the relative orientations of known active ligands not shared by inactives. Its elucidation involves two main stages: (i) the analysis of the training set molecules to identify common pharmacophoric points and (ii) the alignment of the bioactive conformations of these molecules to determine the best overlay of corresponding features [113,119,120]. During this process, molecular features, which are not consistently observed in active compounds should be made optional or removed from the model. Furthermore, spatial constraints can be employed in moieties occupied by inactive compounds and refined to avoid making the model too restrictive. After model refinement, validation studies using statistical metrics must be performed to determine the ability of the model to discriminate between known active and inactive compounds [120].

#### 4.2.3. QSAR

QSAR modeling is widely practiced in industry, government institutions, and universities worldwide. Conceptually, QSAR is a mathematical model describing the relationship between chemical structure and respective biological activity of a set of compounds [121,122,123]. QSAR modeling could be presented as a two-part process. Firstly, chemical structures are converted into a vector of features, which are also known as descriptors and represented generically by the symbol *x* [124]. Thousands of descriptors, both freely available and commercial, encode a compound as a feature vector. Then, machine learning methods (e.g., Random Forest [125], Deep Learning [126], Support Vector Machine [127], etc.) are used to establish quantitative relationships between descriptors and biological activities (represented by the symbol *y*). This involves empirically discovering a function that maps between the feature vectors and activities [124]. Nevertheless, the resulting QSAR model is useful if and only if it is predictive. Hence, the quality of the resulting QSAR model is measured by using the appropriate metrics regarding its ability to correctly predict the activities of compounds experimentally determined [128]. The influence of various factors on QSAR performance decreases in the following row: data quality > molecular descriptors > machine learning approach [129,130,131,132]. Once validated, this model can be applied to untested chemical compounds for numerical prediction of biological activity (continuous model) or the discrimination between active and inactive compounds (binary model) [133].

#### 4.2.4. Quantum Mechanical (QM) Methods

The QM approaches explain the behavior of molecules and its interactions with energy on the scale of atoms and subatomic particles. The increasing popularity of QM methods in CADD is not just a consequence of ever-growing computing power but is also due to their highest accuracy. Both the fast semi-empirical approaches and time-consuming ab initio methods do not suffer from the limitation inherent to the ball and spring description of molecular mechanical (MM) methods and the fixed-charge approximation used in the force fields (FFs). Currently available computing power is not enough for the direct ab initio QM calculations of 3D macromolecules with accuracy like that of in vitro experiments [134]. However, hybrid procedures such as QM/MM free energy simulations, that combine the strengths of both QM (accuracy) and molecular mechanics (MM) (efficiency) methods, have been widely employed to model chemical reactions and other electronic processes in biomolecular systems [135,136,137].

### 4.3. Practical Applications of CADD in Chalcone Field

In the current scenario, design or identification of chalcone derivatives using CADD tools still remain poorly explored. Despite the small number of studies available in the literature, most of them led to the discovery of several lead candidates. Below, we discuss some successful applications of ligand- and structure-based approaches leading to the discovery of promising chalcone derivatives.

#### 4.3.1. Design of Anti-Tuberculosis Agents

**Figure 12:**
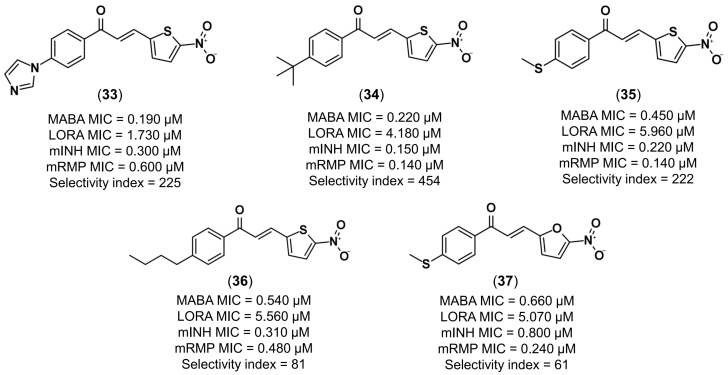
Antitubercular 5-nitro-substituted heteroaryl chalcones prioritized by our QSAR-driven drug design strategy [139].

*Mycobacterium tuberculosis*, the causative agent of tuberculosis (TB), remains a major cause of death around the world. According to the World Health Organization, nearly one-third of the population is infected with tuberculosis, an infectious disease that kills nearly 1.5 million people each year. The current treatment for TB takes approximately six to nine months duration, which normally leads to noncompliance and, hence, the emergence of multidrug-resistant bacteria [138]. Therefore, there is an urgent need for new antitubercular agents that are effective against drug-resistant strains and that reduce the duration of treatment. Aiming at discovering new drugs, our group used QSAR-driven approach to design new series of chalcone derivatives. Initially, we retrieved all chalcone compounds with inhibition data against *M. tuberculosis* H37Rv from the literature. After compilation of the initial dataset and rigorous curation, chalcones were subject to structure-activity relationships (SAR) analysis. Based on these SAR rules, bioisosteric replacements were employed to design new chalcone derivatives with potential anti-TB activity. In parallel, QSAR models were generated using multiple machine learning methods and fingerprints. Using these models, we prioritized 33 compounds for synthesis and biological evaluation [139]. As a result, five 5-nitro-substituted heteroaryl chalcones (see structures **33**–**37** in Figure 12) were found to exhibit MICs at nanomolar concentrations against replicating mycobacteria, low micromolar activity against nonreplicating bacteria, and nanomolar activity against rifampin (RMP) and isoniazid (INH) monoresistant strains (mRMP and mINH, respectively). The series also showed low activity against commensal bacteria and very low cytotoxicity against Vero cells (SI = 61–454). Our results suggest that our designed heteroaryl chalcone compounds are promising anti-TB agents, due to their high potency and selectivity [139].

#### 4.3.2. Discovery of New Tubulin Inhibitors

**Figure 13:**
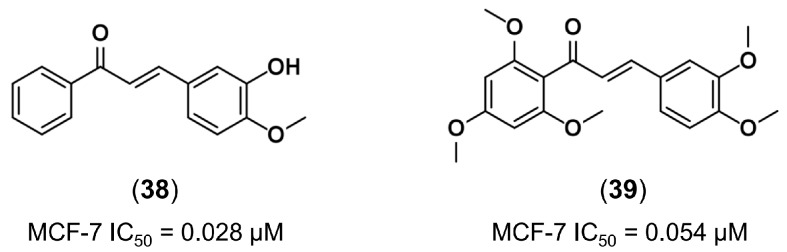
Chalcones with anticancer activity identified by integration of ligand-based pharmacophore screening and molecular docking studies [142].

Microtubules are multifunctional cytoskeletal proteins composed of α- and β-tubulin heterodimers. They have an essential role in regulating cell architectures and functioning as part of the spindle to ensure proper chromosome segregation and cell division. Recently, the clinical use of some tubulin inhibitors, such as taxanes and vinca alkaloids, has been limited by neurotoxicity and drug resistance [140,141]. Therefore, new small-molecule tubulin-binding inhibitors must be developed. Aiming at discovering new drugs, Niu and colleagues [142] integrate LBPs and docking approaches to identify new lead candidates with anticancer activities. Initially, high correlation quantitative pharmacophore models were generated using the SAR of known tubulin inhibitors. Then, an external set composed of 40 compounds with experimental activity data and 800 decoys was used to evaluate the discriminative ability of the models when distinguishing the active compounds from the inactive compounds. After validation, the more predictive model was used in virtual screening of the Specs database, leading to the identification of 952 drug-like compounds (fitted on all the pharmacophoric features) with potential activity. These molecules were subsequently subjected to molecular docking studies to refine the retrieved virtual hits. Finally, five chalcones with diverse substituents and strong molecular interactions with the key amino acids in tubulin binding site were identified. The biological evaluation indicated that compounds **38** and **39** (see Figure 13) showed potent inhibitory activity against MCF-7 cells (IC_50_ of 0.028 µM and 0.054 µM, respectively) [142].

#### 4.3.3. De Novo Design of Histone Deacetylase 2 Inhibitors

**Figure 14:**
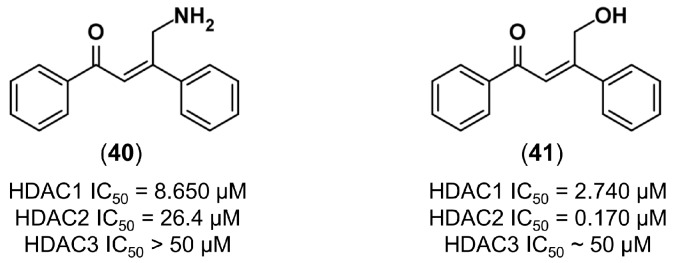
Time-dependent histone deacetylase 2 selective inhibitors identified by integration of quantum mechanics and quantum mechanics/molecular mechanics strategies [147].

Inhibition of histone deacetylases (HDAC) has emerged as a highly promising strategy for the development of new therapeutics against cancer [143,144] and various other human disorders [145]. Although promising, it is particularly challenging to achieve selective inhibitors of the HDAC2 isoform due to its high (97.8%) structural similarities with HDAC1. As a result, it has been very difficult to develop HDAC2 selective inhibitors by employing conventional CADD approaches. Guided by previously characterized HDAC reaction mechanism [146], Zhou and colleagues [147] developed de novo reaction mechanism-based inhibitor design strategy considering reactivity differences between HDAC2 and HDAC1. They worked with the hypothesis that a desired inhibitor should be stable in solution while it should react intramolecularly after binding to the HDAC active site and thus mimics the enzymatic transition state. To examine how designed compounds and the enzyme environment would modulate the reactivity of the intramolecular nucleophilic attack reaction, theoretical calculations on several non-enzyme (QM) and corresponding enzyme models (QM/MM) were carried out. Theoretical reaction barriers indicated that two compounds, β-aminomethyl chalcone (**40**) and β-hydroxymethyl chalcone (**41**), would be stable at the non-enzyme environment, while the intramolecular nucleophilic attack reaction would occur after binding to the HDAC1/2 active site. Computational studies also showed good selectivity of compounds **40** and **41** against HDAC2 vs. HDAC1. Then, β-substituted chalcones were synthesized and tested on a panel of HDACs. In vitro studies showed that β-substituted chalcones preferentially inhibit HDAC1 and HDAC2 and not HDAC3 (Figure 14). Moreover, a distinct time-dependent (~24 h) selective inhibition for the **41** on HDAC2 (IC_50_ = 0.170 µM) against the HDAC1 (IC_50_ = 2.740 µM) and HDAC3 (IC_50_ ~50 µM) was observed [147].

#### 4.3.4. Design of Anti-Leishmanial Agents

**Figure 15:**
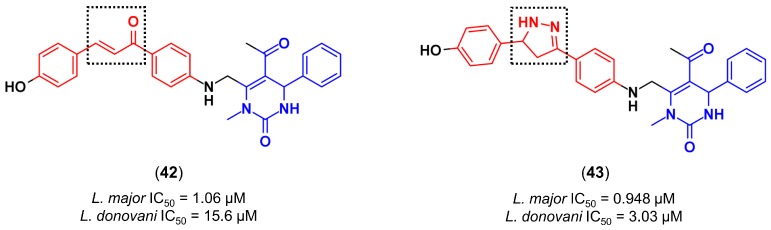
Anti-leishmanial chalcone-dihydropyrimidine hybrids (red and blue, respectively) prioritized by molecular docking studies [151].

Leishmaniasis is an infectious poverty-associated disease caused by trypanosomatid parasites belonging to genus *Leishmania*. In fact, this term includes a wide spectrum of vector-borne diseases with great epidemiological and clinical diversity (e.g., cutaneous, mucocutaneous, and visceral) [148], which are poverty-related and mostly affect the population with the lowest income. Current treatment depends on a limited number of drugs (miltefosine and aminoglycosides) that have issues of toxicity, long-dose regimens, high cost, and the need for parenteral administration [149]. In addition, many of these drugs were developed many years ago, and nowadays various resistant strains exist [150]. Attempting to explore new and more potent anti-leishmanial agents, Rashid and colleagues [151] pursued an in silico-driven design strategy to synthesize novel hybrids with common pharmacophoric features of dihydropyrimidine and chalcone. Dihydropyrimidine based compounds have received considerable attention as potential anti-leishmanial agents due to their inhibition activity on pteridine reductase 1 (PTR1), an enzyme responsible for the salvage of pterins in *Leishmania* parasites [152,153]. Initially, a molecular docking study was carried using the 3D structure of PTR1 from *L. major* aiming at designing a series of hybrids to explore specific interactions of chalcone and dihydropyrimidine moieties in the active site. Based on the binding modes and scores obtained from the docking, authors prioritized 13 hybrid compounds for synthesis and biological evaluation. As a result, low micromolar activities were reported for most of these hybrids. Hybrid **42** displayed the highest activity against *L. major* and *L. donovani* with IC_50_ of 1.06 µM and 15.6 µM, respectively (Figure 15). Finally, bioisosteric replacement of the α,β-unsaturated carbonyl system with a pyrazoline significantly altered the conformation of hybrid **43** (see Figure 15) in active site of PTR1 and increased anti-leishmanial activity (IC_50_ of 0.948 µM and 3.03 µM against *L. major* and *L. donovani*, respectively) [151].

**Figure 16:**
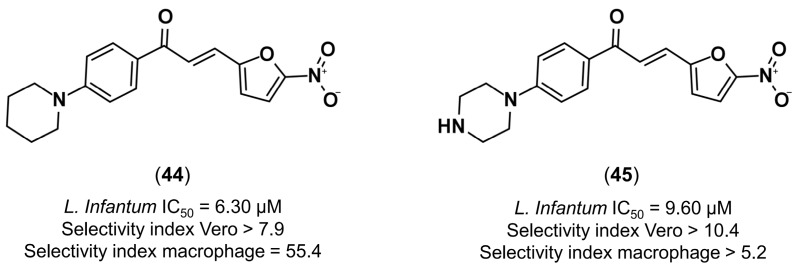
Anti-leishmanial 5-nitro-substituted heteroaryl chalcones identified by integration of pharmacophore screening and molecular docking studies [154].

Our group also employed a computer-aided approach to investigate a set of 32 recently synthesized heteroaryl chalcones as anti-leishmanial agents [154]. Initially, we performed an inverse screening of the best mapping poses of the heteroaryl chalcones against all the pharmacophore models (51.431 LBPs and 16.159 SBPs) available on PharmMapper, a freely accessed web server designed to identify potential target candidates [155]. Then, all primary sequences of the predicted targets were aligned to the *L. infantum* genome. Subsequently, seven potential *L. infantum* targets for the chalcone derivatives were investigated using the underlying assumption that proteins sharing enough similarity (or pharmacophore features) have enhanced probability of sharing the same ligands [156,157]. Due to the absence of X-ray structures on PDB, a homology modeling approach was employed to build the structures of the predicted targets. These structures were then used in docking studies to screen the most promising anti-leishmanial heteroaryl chalcones based on their energy scores. The results of target fishing followed by docking studies suggest that heteroaryl chalcones could act in *L. infantum* because of their interaction with cysteine proteases. Lastly, nine promising compounds and three potentially inactive compounds were experimentally evaluated against *L. infantum* amastigotes and mammalian cells. Two compounds, **44** and **45**, exhibited IC_50_ of 6.30 µM and 9.60 µM against amastigotes, respectively (see Figure 16). In addition, these compounds demonstrated low cytotoxicity towards macrophages (SI > 5.2) and Vero cells (SI > 10.4) [154].

## 5. Conclusions

In summary, the main goal of this review was to emphasize that chalcone derivatives are promising starting points for drug discovery. Chalcone-containing plants have been used for a long time in traditional medical practice. These compounds also have well defined mechanisms of action and potent in vitro and in vivo activities against several pathological conditions. Computer-aided drug design approaches may establish chalcones as a source of inspiration to design and develop novel and more effective drug candidates. Here, we highlighted the significant advances in organic synthesis as well as molecular modification strategies that have contributed to the design of new chalcone derivatives with improved biological activities. Although expert knowledge is indisputably important for successfully guiding drug design, the considerable amount of biological data generated from chalcone derivatives and the public availability of this information has launched the open data era of drug discovery. The availability of these data boost the use of LBDD and SBDD strategies, allowing the generation and validation of computational models for prioritization of chalcone derivatives for synthesis and/or biological evaluation. Despite the small number of examples of in silico-driven design of chalcones available in the literature, most of them have led to the discovery of new lead candidates with potency or affinity values in the low micromolar range. Based on this, we believe that the integration of molecular modification strategies and computational approaches described in this review can accelerate the discovery of new chalcone derivatives that could ultimately enter the market.
